# Development and Testing of an Electronic Diabetes Diary Integrated With a Hospital Information System for Individuals With Type 2 Diabetes Mellitus: Protocol for a Mixed Methods Study

**DOI:** 10.2196/50732

**Published:** 2024-01-23

**Authors:** Geena Skaria, Bhageerathy Reshmi, Sabu K M, Sahana Shetty, Vani Lakshmi R

**Affiliations:** 1 Department of Health Information Management Manipal College of Health Professions Manipal Academy of Higher Education Manipal India; 2 Department of Endocrinology Kasturba Medical College Manipal Academy of Higher Education Manipal India; 3 Department of Data Science Prasanna School of Public Health Manipal Academy of Higher Education Manipal India

**Keywords:** self-management of blood glucose, SMBG, diabetes self-management, DSM, personal health records, electronic diabetes diary, glycemic control, patient adherence, digital health

## Abstract

**Background:**

Type 2 diabetes mellitus (T2DM) is one of the leading noncommunicable diseases that require diabetes self-management (DSM) practices. This study proposes to develop a customized mobile health (mHealth) app integrated with a hospital information system (HIS) to enable real-time, two-way transfer of information between the patient and physician. The captured information in the electronic health record will facilitate physicians to have a chronological account of the patient’s diabetes history and enable tweaking of the treatment.

**Objective:**

The objectives of the study are (1) to develop the HIS-integrated Electronic Diabetes Diary (EDDy) per the end-user expectations at a tertiary care hospital in a south Indian state with a high prevalence of T2DM and (2) to evaluate and test adherence to EDDy in the management of T2DM.

**Methods:**

The study will be carried out in 3 phases. Phase 1 involved in-depth interviews with primary end users to gather information regarding their expectations from the hospital-based EDDy. Phase 2 will use this information to develop a customized mHealth app using an iterative model of software development. Phase 3 will involve a pre- and posttest design; the developed app will be tested among consenting patients, where physicians will receive the patients’ data through the HIS-integrated mHealth app. The pre- and posttest values will be analyzed for adherence leading to improvement in patients’ self-management of blood glucose, user experience, glycemic control, and clinical utility.

**Results:**

Phase 1 was completed on November 28, 2023. Phase 2 commenced in December 2023 and will end in May 2025. Phase 3 will follow afterward.

**Conclusions:**

The proposed app will include a convenient and simple alert system that enables the patient to test glucose values at self-selected intervals, provide grading options to enter diabetic-related complications, enhance patients’ knowledge of tracking and managing the complications of diabetes, and help in maintaining the visual representation of glucose values and complications. The simplicity and usability of the modules are its novelty, which may motivate the patients to keep track of their glucose values and help them attain better health outcomes.

**Trial Registration:**

Clinical Trial Registry India CTRI/2023/03/051077; http://tinyurl.com/4tau4ndb

**International Registered Report Identifier (IRRID):**

PRR1-10.2196/50732

## Introduction

In India, 11.8% of the population have diabetes per the National Diabetes and Diabetic Retinopathy Survey released by the Ministry of Health and Family Welfare in 2019, and this is projected to have a steady rise of 3.8% annually [1]. This prevalence is higher in the southern states and union territories of India [2]. *The Indian Glucose Monitoring Device Market Reports of 2020* [3] suggested that the use of glucometer was always on the rise, and it was specifically noticed that there was an exponential increase in its sale during the COVID-19 pandemic period. Technology-enabled self-management has been increasingly recommended, along with proper medication and lifestyle modification, to tackle diabetes. There are numerous diabetes managements apps available in the Google Play store; however, subscription to such apps is not proportional to the population with diabetes [4]. Literature from other parts of the world suggest that the adoption of digital diabetes-monitoring solutions is expected to reach 21% by 2027 [5].

Diabetes is an irreversible chronic condition that need to be well managed by altering lifestyles, food habits, and physical activities [6]. However, a standard diabetes management regimen may not be feasible to everyone around the globe, as each of these factors varies according to geographical locations and general living conditions [5]. Using customized information that is specific to the local lifestyle may be more effective and easier to ensure adherence. Existing literature suggests that nonadherence to treatment regimen and irregularity in following a diabetes-friendly lifestyle leads to poorly managed diabetes and poor disease outcomes [7]. To enhance the existing measures to manage T2DM, efficient methods needs to be formulated to ensure adherence to treatment regimen [8]. The success of such activities requires active involvement of the patients and caregivers. They should be motivated to follow T2DM management instructions from health care professionals.

This study setting already has an existing system of manual diabetes information documentation that expects its patients to record their self-management of blood glucose (SMBG) practices systematically and submit them to the treating physician during consultation. These glucose monitoring records are used by the physician to evaluate the patient’s glycemic status and make appropriate clinical decisions. This study proposes to automate this process and make it more efficient for the patients and physicians. The proposed electronic diary is expected to reduce the loss of documented values, minimize errors, as well as motivate the patients to effectively make use of technology and manage their condition.

A pilot study was conducted to analyze the knowledge and attitude to digital diabetes management practices among 50 existing patients with diabetes, which indicated that ignorance and technological illiteracy are the main reasons behind nonuse of digital apps among them. Those who used digital apps, such as mobile health (mHealth) apps, suggested that the information available is more general in nature and that they find it difficult to navigate and comprehend the information, eventually leading to the discontinuation of its use.

## Methods

### Overview

The study will be carried out in the outpatient department (OPD) of the endocrinology department at a tertiary care hospital in a south Indian state, and it is divided into 3 phases. In phase 1, a qualitative study was conducted; in phase 2, the mobile app with be developed and integrated with a health information system (HIS); and finally, in phase 3, the developed app will be tested for its feasibility using a pre- and posttest design.

The study is based on the principles of the “theory of change,” where stakeholders will be consulted through in-depth interviews (IDIs) to identify the deterrents to acceptance and adherence of electronic diabetes-monitoring practices, potential challenges, as well as ethical issues related to electronic information sharing. This feedback will provide the cues for the development of a customized electronic diabetes-monitoring system. The study would also attempt to identify the reasons that would lead to the discontinuation of electronic monitoring practices among patient with T2DM and address them by developing a more user-friendly mHealth app. This study will also analyze the patient acceptance of the mHealth app and their adherence to SMBG practices.

The model will then be developed and integrated with an HIS. Finally, it will be tested among patients with T2DM regarding their acceptance and adherence to using the Electronic Diabetes Diary (EDDy), its usability, contextual relevance according to user expectation, and clinical utility in terms of glycemic control.

### Ethical Considerations

This study has been approved by the Institutional Ethics Committee (IEC; IEC No:223/2022). The participants personal information will be anonymized, and all privacy, confidentiality, and compensation policies will be adhered to as per the IEC guidelines.

### Pilot Study

As a precursor to this study, a pilot study was conducted in a tertiary care hospital among 50 patients with T2DM to understand the existing personal record-keeping practices, the challenges they face, and their awareness of personal record keeping. The perception, expectations, and concerns of the target population were studied using a validated questionnaire survey. Patients with T2DM who are within the age group of 40-65 years were included in the study. The study setting encouraged patients with T2DM to document their SMBG values by providing a data sheet to enter the values and asking patients to bring it with them when they come for a doctor’s appointment. It is a valuable tool for the doctor to assess the values and make better clinical decisions in the management of the disease.

A cross-sectional study was conducted, and a purposive sampling technique was followed to select the participants. A mixed-type questionnaire (both open- and close-ended questions) was used to collect data.

### Phase 1

#### Study Design

IDIs with key stakeholders was carried out to identify the reasons for nonadherence, expectations from EDDy, and mandatory requirements to develop the intervention mechanism. The key stakeholders included the following participants:

- Patients with T2DM
- Their caregivers
- Treating doctors
- Diabetes counselors (includes nursing counselors, diet counsellors, and physiotherapists)

#### Sample Selection

The IDI participants were enrolled according to the following criteria: patients who own an Android smartphone and those who regularly attend doctor’s appointments (ie, those who visit the OPD at least once in the last 6 months). Patients who are literate, can understand the local language (Kannada or Tulu) or English, aged 40-65 years, and have hemoglobin A_1c_ values between 7% and 10% were selected to ensure homogeneity. Patients with gestational diabetes and other terminal illnesses were excluded. The IDIs were conducted among the immediate caregivers of the patients (ie, those who are actively involved in the care of the patient). To understand the practical aspects of the treatment and management of patients, physicians, nurses, and counsellors were also interviewed.

Even with the availability of various diabetes management apps, studies suggest they are not very popular among the general population. The reason for this underuse and nonadherence, along with their expectations from mHealth apps, are the key areas that were addressed among patients with T2DM and their caregivers. The doctors and diabetes counselors were interviewed to understand the challenges they face while managing patients with T2DM and their SMBG practices.

#### IDI Process

**Figure 1:**
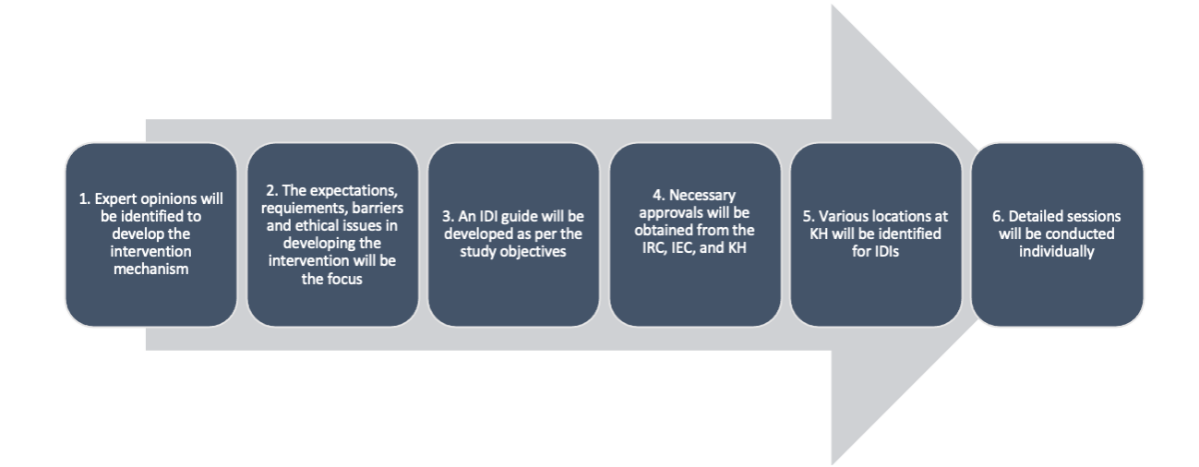
Process of the in-depth interviews (IDIs). IEC: Institutional Ethical Committee; IRC: Institutional Research Committee; KH: Kasturba Hospital.

The IDIs focused on the perspectives of patients with T2DM and their caregivers along with physicians and diabetes counselors who are actively involved in the care of patient with T2DM. Additionally, IT and health information management professionals were also consulted on ethical challenges and technical expertise. The IDI guide was designed based on the objectives of the study and the content was validated by experts. The IDI process is shown in Figure 1.

Qualitative data generated during the IDIs will be analyzed by using ATLAS.ti software (version 8; ATLAS.ti), and a report will be prepared. Based on the report, the researchers and the subject experts will identify the most feasible options that will be considered for incorporation into EDDy.

### Phase 2

#### Development of EDDy

Phase 2 is the main part of this research, where the software will be developed based on inputs from the IDIs and the literature. The mobile app will be developed on Android Studio (Google) using an iterative model design.

**Figure 2:**
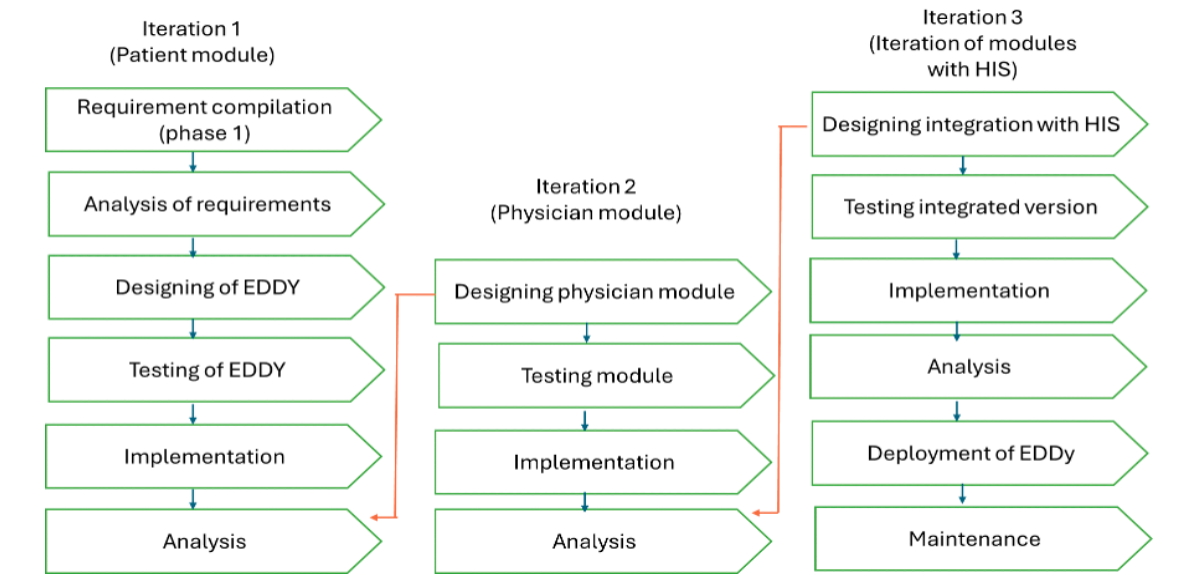
Iterative model of Software Development Life Cycle. EDDy: Electronic Diabetes Diary; HIS: hospital information system.

The iterative model (Figure 2) is one of the Software Development Life Cycle models, where the development of a system goes through repeated cycles (iterative) and is conducted in smaller portions at a time (incremental) [9]. As and when the different modules of the app are developed, they will be tested and reviewed against the end user’s expectation, and accordingly, modifications and further development will be done. This process will enable easier debugging and fixing of errors as and when tested.

The development and testing of the app will involve development, pilot-testing, and debugging at each phase. Each iteration will be subjected to the development, testing implementation, and analysis of each component of EDDy. The components for incorporation into the software will be validated by the following experts:

- An endocrinologist
- Qualitative experts
- The IT team at the hospital

The expected pattern and time period for phase 2 are as follows.

#### System Development

##### Step 1: Requirements

This is the phase where the stakeholders’ requirements are enquired and compiled, which was primarily covered in phase 1. The captured data will be systematically entered into Microsoft Excel for further evaluation.

##### Step 2: Analysis

The compiled information will be divided into feasible and nonfeasible items as per the system development and HIS-interfacing requirements. This step will be conducted with the researchers and technical experts.

##### Step 3: Designing and Development

This step will be done by a software developer with the support of a researcher. Once the feasible requirements are sorted and finalized by the researcher, the design of the app will take place. First, a prototype of the system will be developed, and the requirements for each module of the app will be determined. This step includes the coding of the app based on the design. Android Studio, an open-source program for Android software development, will be used to develop the proposed mobile app. The iteration process has 3 phases, where a patient module and a physician module will be designed, and integration with an HIS will be performed as depicted in Figure 2.

##### Step 4: Implementation

After development, each module of EDDy will be pilot-tested for its intended use. The modules will be connected to a web-based backend database application to help the validation of content, visualization, and analysis of the data collected.

##### Step 5: Testing and Quality Assurance

The developed app will undergo a standard testing and quality assurance process to identify and rectify any issues and bugs. Each module will be validated during testing to ensure its intended performance.

##### Step 6: Analysis of Feedback

A pilot test among 2 potential users will be carried out at each level of iteration, and user experience will be measured using a Likert scale on various aspects of the app’s functionality. The parameters that will be measured during the piloting process will be the app’s functionality, usability, accessibility, compatibility, and performance under real-life scenarios.

Once the app is fully developed and interfaced with an HIS, it will be installed in the respective systems of the stakeholders and tested for its performance. Gaps and technical glitches will be rectified at each phase.

##### Step 7: Deployment

The final app will be deployed among end users (patients and physicians).

##### Step 8: Maintenance

The final app will be integrated into an HIS. Once the module is fully functional, the study will proceed to phase 3.

#### Expected Outcomes From EDDy

The app is expected to improve the SMBG practices in patients with T2DM. EDDy is expected to motivate the use of the app (*acceptability*) and improve the user experience on the *contextual relevance*. The *ease* of using the customized system and a clinician’s feedback mechanism through the physician module is expected to improve the *clinical utility* of the app. It is expected that adherence to using the app would improve compliance to treatment protocol, thus improving treatment outcomes.

The entry of information into EDDy can be done either by the patient or their caregiver. The entry of SMBG values by the patient is a standard clinical practice in patient management. The current practice is that these self-monitored blood glucose values (SMBG data) is maintained by the patient on a printed form provided by the hospital. It is standard practice that such SMBG data function as a mechanism that helps physicians to optimize medication in routine clinical practice [10].

**Figure 3:**
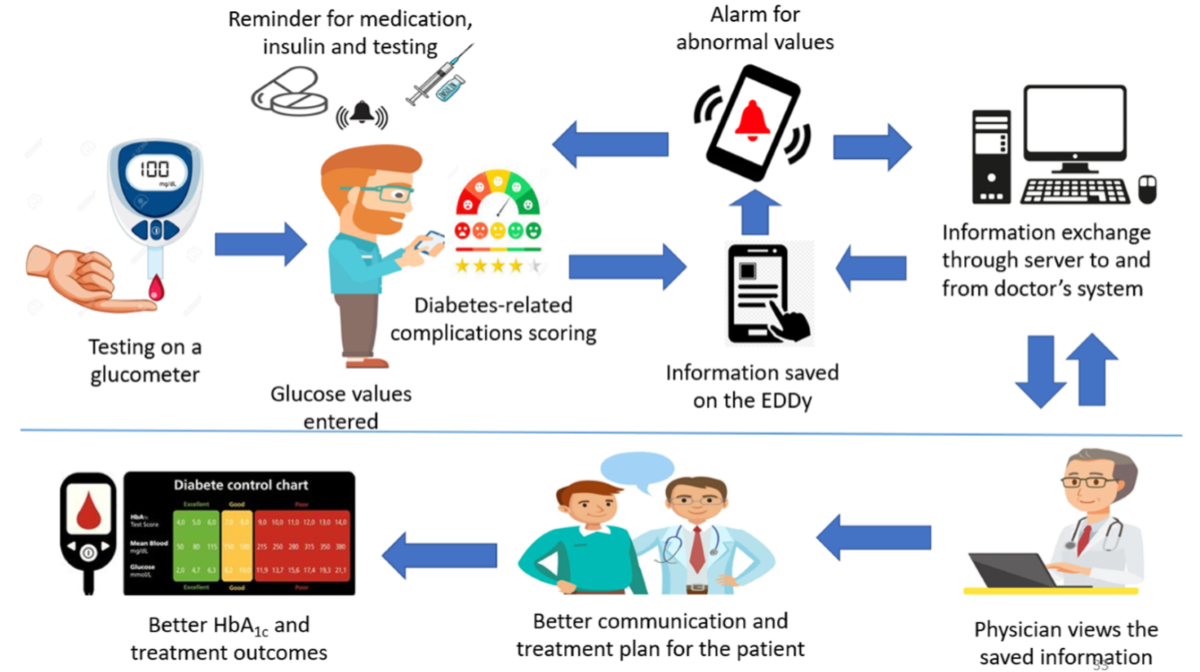
Prototype of EDDy.

A researcher will provide instruction to the patient or patient party on how data entry needs to be done on the app. Additionally, a built-in user manual will be provided with the system. This will be developed as a part of the app development. A multilingual *user manual* or *instructional videos* will be developed to assist the patient in using EDDy. The pictogram in Figure 3 represents the workflow of EDDy.

### Phase 3

#### Study Design

Phase 3 will involve testing the adherence and usability of the developed app. This phase will use a pre- and posttest design among 48 patients with diabetes selected through purposive sampling.

At a 5% level of significance with 80% power and an effect size of 0.25, the minimum sample size required for a single-group, repeated measures study design involving 1 primary quantitative outcome (adherence) measured across 3 time points (preintervention, after 3 months, and after 6 months; correlation among repeated measures is 0.3) to understand the adherence to EDDy integrated with an HIS is 38 individuals. Incorporating a dropout rate of 20%, the minimum sample size requirement is 48 individuals.

The sample size was computed using G*Power (version 3.1.9; Universität Düsseldorf). The sample size may be amended (if required) based on pilot study findings.

The study setting will be the endocrinology OPD of a tertiary care hospital in coastal Karnataka, India.

Regarding study participants, patients with T2DM who have regular follow-up records will be considered for the study.

This study will analyze the adherence of patients with T2DM to EDDy in comparison to the existing manual method of documentation. Adherence will be evaluated in terms of the following:

- Adherence of entering SMBG values as predetermined by the patient: The study participants will be instructed to fix the frequency that they will monitor their blood glucose; based on this frequency, they will be prompted to conduct the glucometer testing and enter the values into EDDy. The values will be stored in the app and will be transferred to the server on a regular basis as the EDDy is integrated with an HIS.
- Adherence to diabetes self-management (DSM) practices: Pre- and posttest adherence to DSM practices will be analyzed using the same content-validated questionnaire.
- Adherence to routine blood investigations and screening regimen: Pre- and posttest adherence to periodic blood investigation and comorbidity screening will be analyzed.

The inclusion and exclusion criteria for sample collection will remain same as that of phase 1.

Phase 3 will be evaluated in 3 intervals:

- First evaluation (preintervention): During enrollment into the study, the patients will undergo an entry survey using a content-validated questionnaire to analyze the current DSM and SMBG practices they follow.
- Second evaluation (follow-up): An interim follow-up will be conducted in the third month.
- Third evaluation (postintervention): The same questionnaire used in the first evaluation will be used to evaluate DSM and SMBG practices after the intervention.

**Figure 4:**
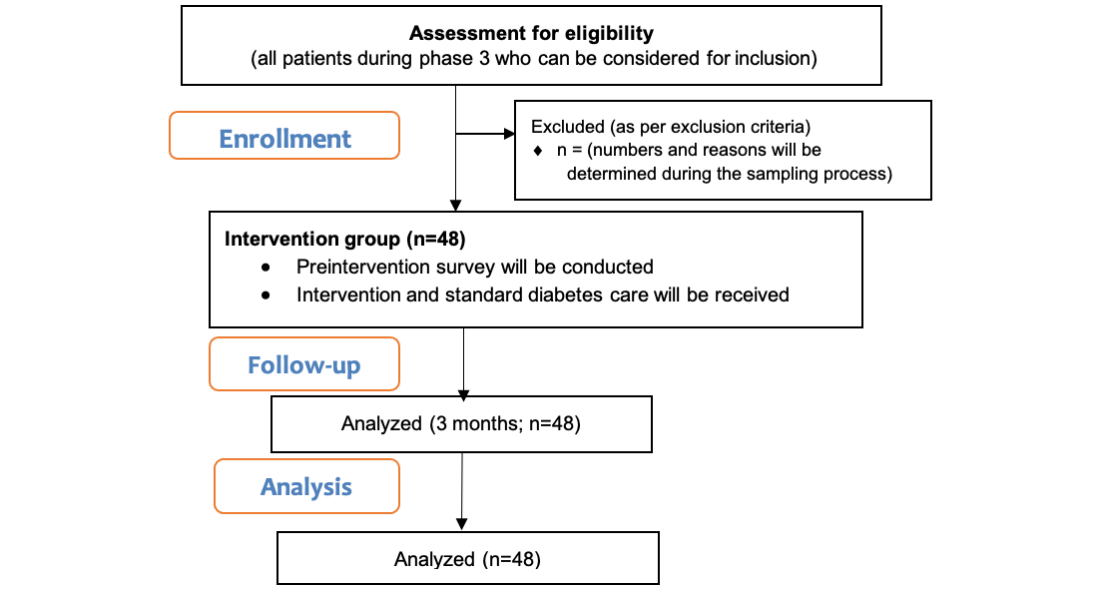
Pre and Posttest Process of development of EDDy.

The study participants will be provided with EDDy on their mobile phones and will be trained to use it along with standard diabetes care. Training materials will be a part of the app for patient’s ready reference. The pre- and posttest process is shown in Figure 4.

#### Analysis

Study participants will be followed up for 6 months and will be analyzed primarily for their adherence to using EDDy. It will be assessed based on patients’ willingness to use it, the ease of use, their acceptance of EDDy in terms of the contextual relevance, and the clinical utility. The participants will undergo an exit survey at the end of the study period using a validated questionnaire. It will be specific for assessing the qualitative parameters such as *user experience*, *glycemic control*, *adherence leading to improvement in their SMBG*, and *clinical utility.*

The qualitative outcomes are as follows:

- Ease of use and adherence: User’s perception on accessing the various modules in the app, ease of entering information, ease for retrieving information, and ease of comprehending information
- Glycemic control: Adherence in terms of regular monitoring and tracking of blood glucose values to keeps it under check, leading to well-managed T2DM
- Contextual relevance: Relevance of the contents of EDDy in terms of the user expectations and requirements as expressed in phase 1 of the study
- Adherence to SMBG and DSM practices: Reminders and alerts that ensure adherence to medications, insulin injections, regular glucose testing, and comorbidity screening
- Clinical utility: Improvement in treatment outcomes due to adherence to reminders or alerts and monitoring of blood glucose, adherence to medication, and follow-ups

The frequency will be tested based on the Diabetes Co-Conditions Screening Checklist provided by the Association of Diabetes Care & Education Specialists [11].

The following standard tools will be used for assessment, which are modified according to the study requirements and will be content validated by experts:

- Perceived Health Web Site Usability Questionnaire: A 12-item questionnaire that is validated for assessing the usability of health websites for older adults will be used [12].
- Diabetes Self-Management Questionnaire: It describes self-care activities related to diabetes. SMBG practices will be analyzed using this tool. A validated 12-item questionnaire to assess self-care activities associated with glycemic control will be used [13].
- A self-developed, content-validated questionnaire: Adherence will be evaluated, including the regularity of use; ease of use with respect to entering diabetes-related information such as regular testing and entry of glucose levels; adherence to medications or insulin doses; reporting of diabetes-related emergencies or complications, if any; and adherence to periodic screening such as laboratory investigations, peripheral neuropathies, retinopathy, diabetic foot, and BMI.

The physicians will be interviewed to assess the clinical utility of the app and their experience in using the app using a content-validated questionnaire.

#### Expected Outcomes

A customized mHealth app that provides adequate information specific to the T2DM management will be developed. This can lead to an effective intervention that will reduce the risk of a person diagnosed with T2DM from developing further disease complications, such as chronic kidney disease, diabetic foot, retinopathies, and peripheral neuropathies, thereby reduce the cost of health care expenditure.

## Results

### Pilot Study

Out of 50 patients with T2DM (aged 40-50 years), 22 (44%) documented their self-monitored glucose values, whereas the remaining 28 (56%) did not. The percentage score of sources used by patients with diabetes to document the values of self-care activities showed that most of the patients (n=21, 42%) were using a personal diary to document, whereas only 1 (2%) patient each used a diabetes app and mobile notes. Responses from patients with diabetes regarding the necessity of personal health information in diabetes management showed that 31 (62%) patients were not completely aware of personal health information management.

The study results reflected the perception of patients with T2DM about their personal health information documentation practices. Additionally, the study gave a brief outlook about the challenges and barriers that patients face during their DSM activities.

### Study Status

Phase 1 was completed on November 28, 2023. Phase 2 of the study, which is the app development, commenced in December 2023 and will end by March 2025. The wireframe model of EDDy is shown in Multimedia Appendix 1. Phase 3 of this study will follow afterward, analyzing the effectiveness of adherence to the app and its usability.

## Discussion

### Pilot Study Findings

It was evident from the pilot study that there is a strong requirement to promote documentation of SMBG and DSM practices. Overall, the study concluded that there is a scope for strengthening DSM, knowledge development, and improved self-documentation practices by promoting personal health information practices among patients with diabetes. It has the potential to improve the quality of life of patients with diabetes by better promoting self-care.

### Expected Findings

When considering a country such as India, which has a very diverse population belonging to different socioeconomic backgrounds, it is difficult to implement a standard practice to tackle a problem. Statistics show that out of 45% of Indians over 45 years of age, about 11.5% are diagnosed with diabetes or high blood sugar levels [3]. Patients belonging to this age group are comparatively less familiar with technology and digital apps. Understanding their requirements and developing a hospital-based electronic diary may promote its use and adherence among them and, eventually, all patients with T2DM.

Considering India’s socioeconomic background, the requirements for this population need to be assessed from various angles. It can be demonstrated by analyzing the findings of the study conducted by Walle et al [14] among 422 patients with diabetes in Ethiopia. The study aimed at understanding the willingness of patients with diabetes mellitus to use mHealth app and its associated factors for self-care management in a low-income country as a precursor to digital health implementation in Ethiopia. The study concluded that the mHealth app developers should consider factors such as the patients’ age, place of residence, internet connectivity, attitude, perceived ease of use, and perceived usefulness while developing similar apps [14].

The pilot study conducted to analyze the awareness of patients with T2DM also clearly stated that they were aware of the importance of SMBG and DSM practices and the availability of mHealth apps to help them manage their T2DM. However, they were not very keen on using it due to various personal factors.

It was evident from the findings that there is a clear requirement to generate awareness about T2DM management and the use of mHealth apps. It is equally important to understand the challenges that keep them from using available mHealth apps. Based on these observations, if an mHealth app can be developed and the information can be transferred to the patients’ records in real time, it will be beneficial for both the patients and the doctors to better manage the condition.

### Conclusion

The literature review and pilot study conducted suggests that an easily manageable, personalized diabetes-monitoring system for patients to do regular self-checks and that provides the end users with accurate data on the patient’s diabetic history will be beneficial for better management of T2DM. It would enable better sharing of information between patients and doctors, thus improving the communication between patients and doctors, which is significant in the management of diabetes and leads to better treatment outcome. The available apps are not very popular among the end users as they find it difficult to use and understand the contents. To ensure better usability and adherence, a convenient and simple alert system that enables the patient to conduct glucose testing at intervals set by the patients themselves can be designed. The proposed app EDDy will provide grading options to enter the diabetic-related complications (such as neuropathy, retinopathy, diabetic ulcers, etc), thus alerting patients and doctors for prompt action. It is also expected to enhance patients’ knowledge of tracking and managing the complications of diabetes and help in maintaining the visual representation of glucose values and complications. The simplicity and usability of the module are its novelty, which may motivate the patients to keep track of their glucose values and help them to attain better health outcomes.

## Appendix

Multimedia Appendix 1 Wireframe model of Electronic Diabetes Diary (EDDy).

## Abbreviations

CTRI: Clinical Trial Registry of India
DSM: diabetes self-management
EDDy: Electronic Diabetes Diary
IDI: in-depth interview
IEC: Institutional Ethics Committee
HIS: hospital information system
mHealth: mobile health
OPD: outpatient department
SMBG: self-management of blood glucose
T2DM: type 2 diabetes mellitus

## Data Availability

As this is a study protocol, the results of the pilot study have been reported, and the data will be submitted as required.
