# Supplementary material for: Development and Testing of an Electronic Diabetes Diary Integrated With a Hospital Information System for Individuals With Type 2 Diabetes Mellitus: Protocol for a Mixed Methods Study
Source: JMIR Res Protoc. 2024 Jan 23;13:e50732. doi: 10.2196/50732 (PMC10848138; doi:10.2196/50732)

Wireframe model of EDDy

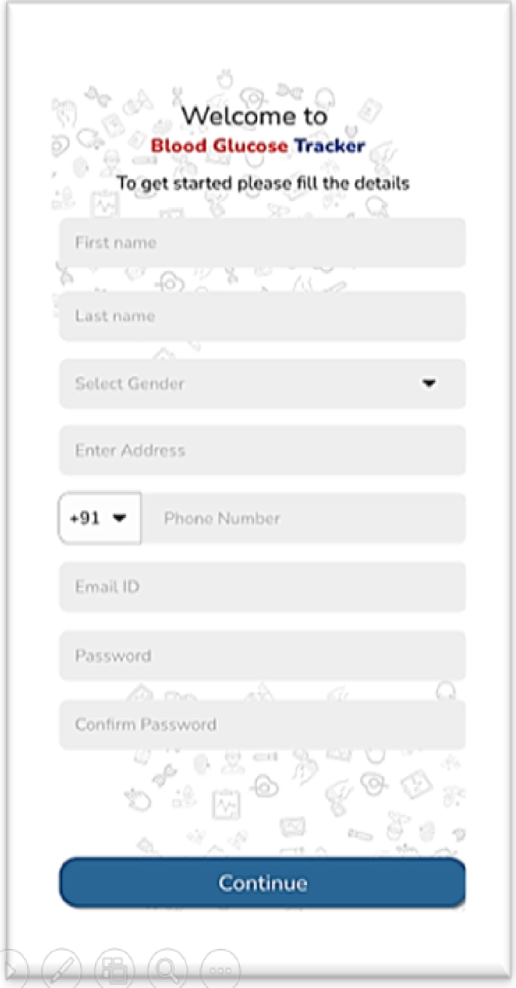

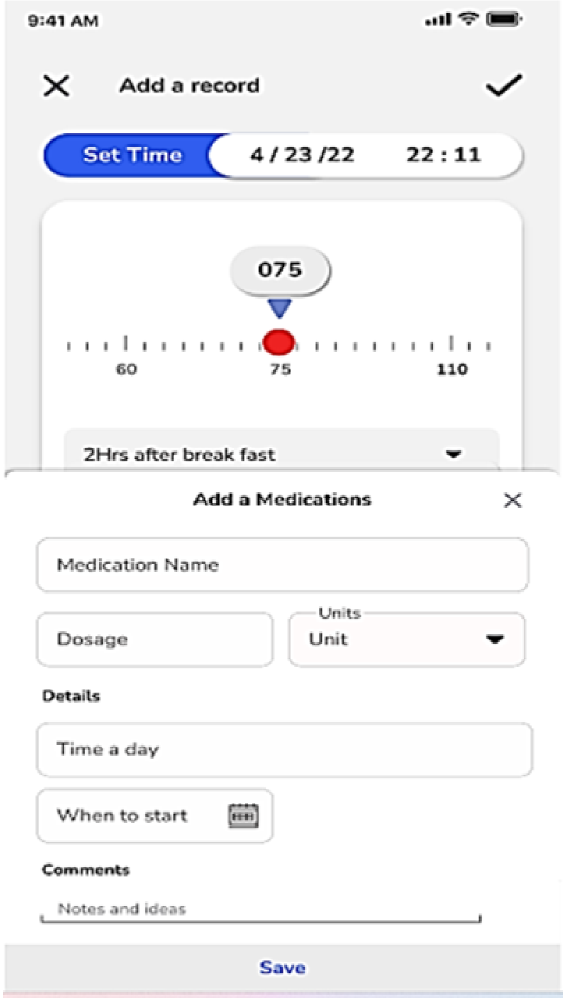

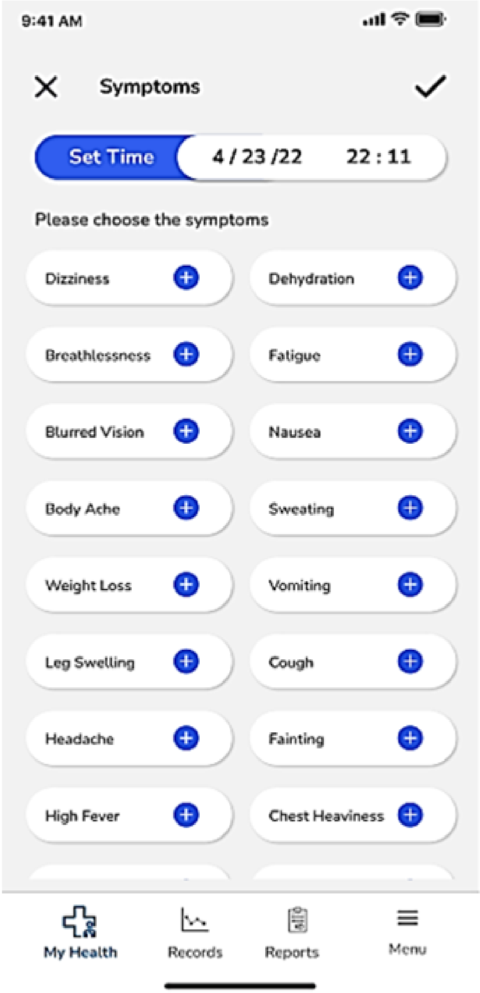

Supplement: Multimedia Appendix 1 [file resprot_v13i1e50732_app1.pdf]
